# ^68^Ga-HER2 PET/CT for Predicting Pathologic Response to Neoadjuvant Therapy at Early Treatment and Preoperative Stages in HER2-Positive Breast Cancer: A Prospective Study

**DOI:** 10.2967/jnumed.125.271529

**Published:** 2026-10

**Authors:** Sinuo Gao, Zhaoqi Zhang, Chao Shi, Cheng Cheng, Xindi Ma, Xinming Zhao, Yunjiang Liu

**Affiliations:** 1 Department of Breast Center, Fourth Hospital of Hebei Medical University and Hebei Provincial Tumor Hospital, Shijiazhuang, China;; 2 Department of Nuclear Medicine, The Fourth Hospital of Hebei Medical University and Hebei Provincial Tumor Hospital, Shijiazhuang, China; and; 3 General Surgery, The First Hospital of Qinhuangdao, Qinhuangdao, China

**Keywords:** HER2-positive breast cancer, ^68^Ga-HER2 PET/CT, NAT, pCR

## Abstract

This study aimed to evaluate the predictive performance of ^68^Ga-HER2 PET/CT in assessing pathologic response to neoadjuvant therapy (NAT) in human epidermal growth factor receptor 2 (HER2)–positive breast cancer. **Methods:** In total, 45 patients with newly diagnosed HER2-positive breast cancer eligible for NAT were enrolled. All participants received at least 4 cycles of trastuzumab combined with pertuzumab or pyrotinib-based therapy, followed by surgery. ^68^Ga-HER2 PET/CT was performed at baseline (PET1), at the end of the second therapy cycle (PET2), and before surgery (PET3). The SUV_max_ of primary breast tumors and metastatic lymph nodes were recorded. **Results:** Pathologic complete response (pCR) was achieved in 31 patients (70%), whereas 13 (30%) showed non-pCR. The SUV_max_ at PET1 did not differ significantly between pCR and non-pCR groups (3.76 ± 1.97 vs. 3.79 ± 2.71, *P* = 0.349). However, the SUV_max_ at PET2 and PET3 were significantly lower in the pCR group compared with the non-pCR group (PET 2: 0.45 ± 0.71 vs. 2.01 ± 2.36, *P* = 0.008; PET3: 0.12 ± 0.39 vs. 1.67 ± 2.94, *P* = 0.014). The optimal cutoff values for predicting pCR included an SUV_max_ of no more than 1.15 (area under the curve [AUC] = 0.738) at PET2 and no more than 0.50 (AUC = 0.735) at PET3 and a ΔSUV_max_ of at least 61% (AUC = 0.742) at PET2 and at least 72% (AUC = 0.743) at PET3. Application of the PET2 cutoff thresholds for SUV_max_ and ΔSUV_max_ identified a therapy-sensitive subgroup, which had a positive predictive value for pCR of 85%—representing a 15% higher absolute likelihood of pCR compared with the nonsensitive subgroup—while the method provided a negative predictive value of approximately 60%. **Conclusion:** Dynamic changes in ^68^Ga-HER2 PET/CT uptake during NAT provide an effective early indicator of therapeutic sensitivity in HER2-positive breast cancer, supporting its value for predicting pathologic response at both early treatment and preoperative stages.

## Visual Abstract

**Figure:**
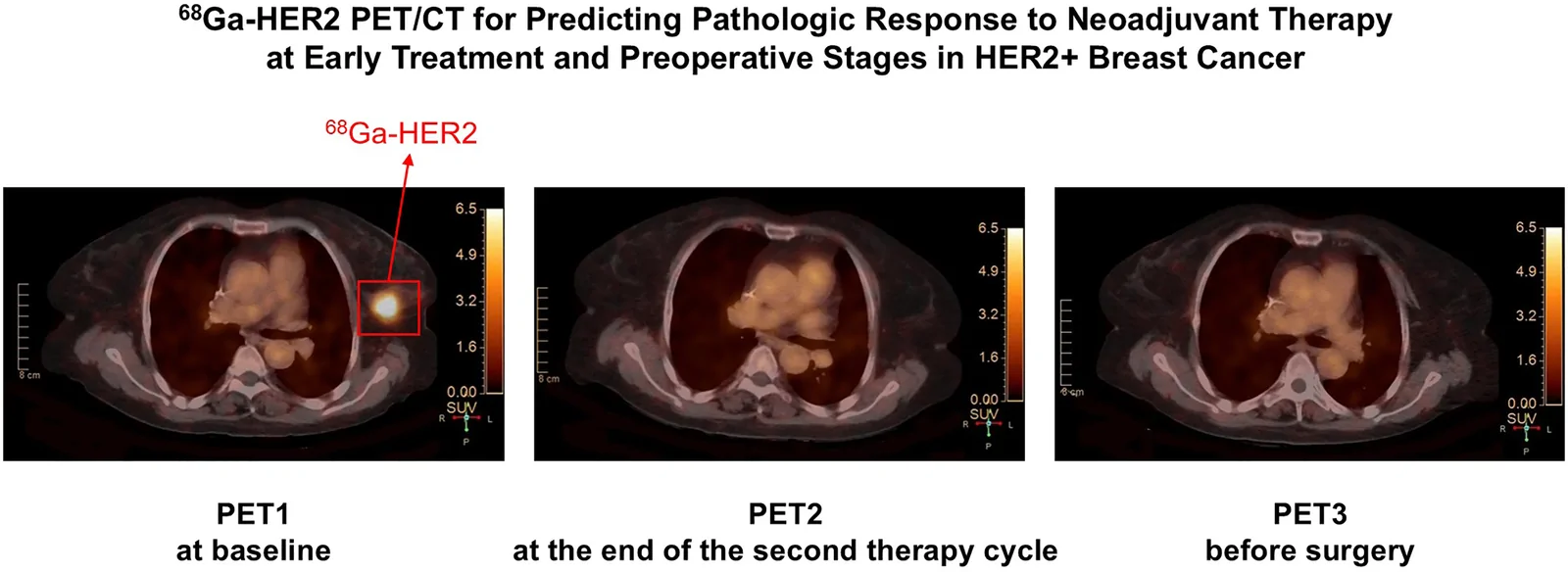

Breast cancer, with 2.296 million new cases reported in 2022, remains the leading cause of cancer-related morbidity and mortality in women (1). Although advances in screening and therapy have improved outcomes, human epidermal growth factor receptor 2 (HER2)–positive tumors (20%–25% of cases) present unique challenges because they are aggressive and associated with poorer survival rates (2). Although targeted agents such as tyrosine kinase inhibitors and antibody–drug conjugates have improved management, resistance and toxicity persist. Neoadjuvant therapy (NAT) combining chemotherapy with dual HER2 blockade enables tumor downstaging, in vivo drug sensitivity assessment, and surgical optimization.

The evaluation of NAT efficacy relies on pathology, imaging, and clinical examination. Pathologic assessment provides definitive pathologic complete response (pCR) determination but suffers from delayed feedback and sampling limitations (3,4). Imaging modalities each have distinct advantages and drawbacks: ultrasound offers cost-effective assessment of lymph nodes but is operator-dependent (5); mammography detects calcifications well but poorly measures tumor size (6,7); and MRI, the standard NAT monitoring modality in multinational guidelines, faces challenges in apparent diffusion coefficient measurement and differentiation between fibrosis and tumors (8). Clinical examination, although rapid, lacks accuracy. This trade-off between accuracy and timeliness drives the need for novel approaches.

PET/CT combines functional and anatomic imaging. Although ^18^F-FDG PET/CT is used in diagnosis and staging, its NAT response assessment is limited by false positives and a failure to capture molecular heterogeneity (9). For instance, HER2-positive tumors may show transiently elevated uptake after treatment, whereas luminal cancers may not promptly reflect metabolic changes (10). These limitations necessitate more specific molecular probes.

^68^Ga-HER2 PET/CT has emerged as a promising molecular imaging modality for the noninvasive, whole-body visualization of HER2 expression. This technique utilizes a targeted probe composed of a HER2-specific affibody (58 amino acids), a DOTA chelator, and the radionuclide ^68^Ga (11). The affibody component, with a molecular weight of approximately 7 kDa, offers high affinity and selectivity for HER2 receptors along with favorable in vivo stability (12). These properties contribute to rapid systemic clearance and efficient tumor penetration, distinguishing it from conventional antibodies. Another key advantage lies in the use of ^68^Ga, which, with a 68-min half-life, can be produced on demand from a portable generator and delivers a relatively low radiation dose, facilitating serial imaging sessions (13). Despite these advantages, evidence regarding its utility in assessing the efficacy of NAT for breast cancer remains scarce.

The present study was designed to determine the predictive significance of ^68^Ga-HER2 PET/CT in evaluating NAT responses at an early stage in HER2-positive breast cancer, aiming to establish a more accurate approach for therapeutic response assessment in this patient population.

## MATERIALS AND METHODS

This single-center, prospective, exploratory study was designed to evaluate the predictive performance of ^68^Ga-HER2 PET/CT for pCR to NAT in HER2-positive breast cancer. All participants received at least 4 cycles of trastuzumab combined with pertuzumab or pyrotinib-based therapy, followed by surgery. ^68^Ga-HER2 PET/CT scans were performed at 3 predefined time points: (1) at baseline before the initiation of NAT (PET1) to establish the initial tumor profile; (2) at the end of the second therapy cycle (PET2) to assess early therapeutic response for early prediction of treatment efficacy; and (3) after completion of therapy and before surgery (PET3) to serve as the final imaging assessment for direct correlation with the pathologic gold standard (pCR) and to evaluate the extent of any residual disease before surgery. The primary endpoint was the predictive value of ^68^Ga-HER2 PET/CT for pCR. Secondary endpoints included the sensitivity, specificity, positive predictive value (PPV), and negative predictive value (NPV) of ^68^Ga-HER2 PET/CT in assessing treatment response. The study flowchart is presented in Figure 1.

**FIGURE 1.**
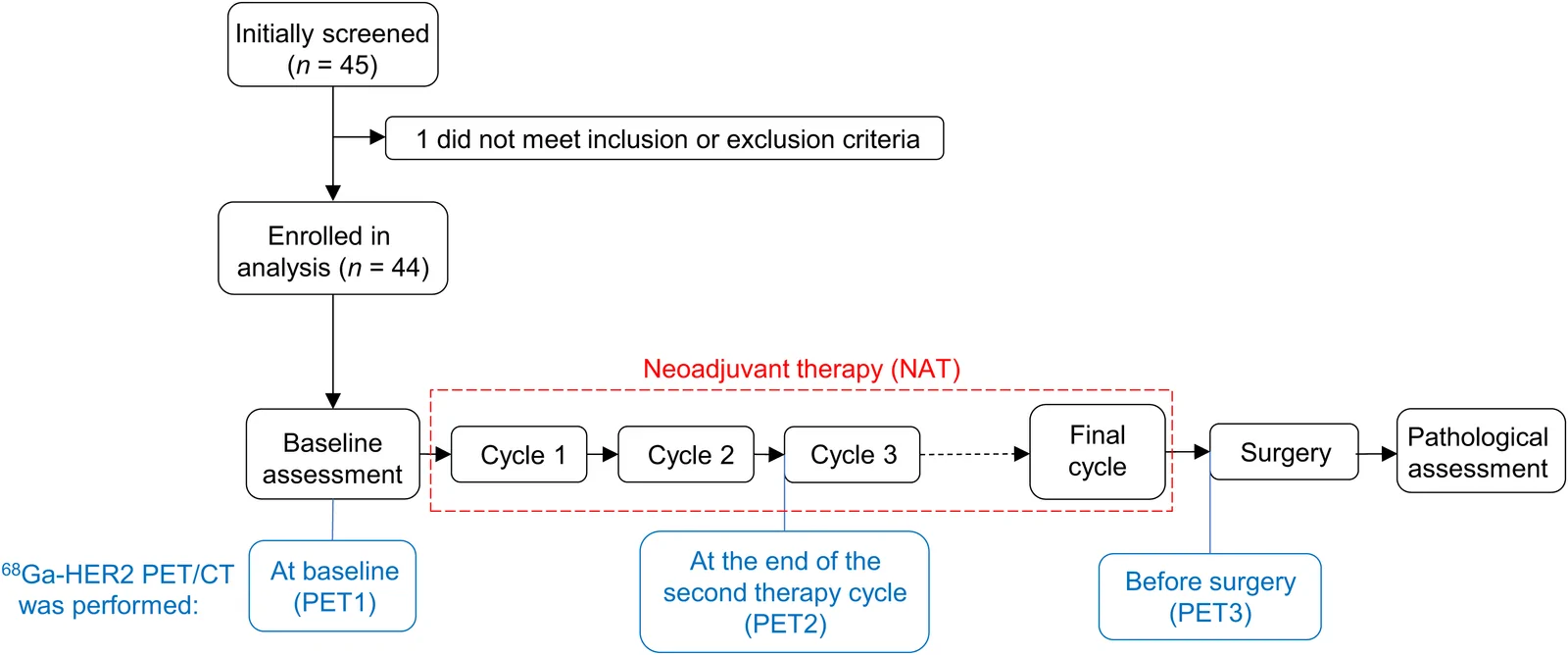
Study flowchart.

### Patients

The study was approved by the Ethics Committee of the Fourth Hospital of Hebei Medical University (approval 2022114) and registered with ClinicalTrials.gov (NCT04499365). All participants provided written informed consent before study enrollment. Between June 2023 and August 2024, 45 patients with newly diagnosed HER2-positive breast cancer who were candidates for NAT were recruited; 44 satisfied all criteria and completed the protocol. Eligibility required an age of 18 y or older, stage II–IIIC HER2-positive breast cancer, receipt of at least 4 cycles of trastuzumab combined with pertuzumab or pyrotinib-based NAT, and subsequent surgical resection on completion of treatment. Exclusion criteria included pregnancy or lactation, the presence of severe comorbidities, an inability to tolerate systemic therapy or surgery, and distant metastatic disease.

As this was a prospective exploratory study, no prior data were available for sample size calculation. The sample size was determined on the basis of feasibility, aiming to assess preliminary trends and the efficacy of ^68^Ga-HER2 PET/CT in predicting pCR to NAT, which will inform future confirmatory clinical trials.

### Imaging Assessment

#### ^68^Ga-HER2 PET/CT

All participants underwent ^68^Ga-HER2 PET/CT. The PET1, PET2, and PET3 scans were performed on the same PET scanner using identical protocols for image acquisition, reconstruction, and quantitative analysis. Imaging was acquired 50 min after intravenous administration of 111–185 MBq (3–5 mCi) of ^68^Ga-HER2. The SUV_max_ of each lesion was independently measured by 2 experienced PET/CT radiologists, both holding senior professional titles (associate chief physician or above) and masked to patients’ clinical information; any discrepancies were adjudicated by a third senior radiologist. ΔSUV_max_1 and ΔSUV_max_2 were calculated using Equations 1 and 2, respectively:

$$\Delta {\mathrm{SUV}}_{\max}1=\frac{\mathrm{PET}2{\;\mathrm{SUV}}_{\max}-\mathrm{PET}1{\;\mathrm{SUV}}_{\max}\;}{\mathrm{PET}1\;{\mathrm{SUV}}_{\max}}\times 100\%,$$ Eq. 1

$$\Delta {\mathrm{SUV}}_{\max}2=\frac{\mathrm{PET}3{\;\mathrm{SUV}}_{\max}-\mathrm{PET}1{\;\mathrm{SUV}}_{\max}\;}{\mathrm{PET}1\;{\mathrm{SUV}}_{\max}}\times 100\%.$$ Eq. 2

#### MRI

During NAT, all patients underwent breast MRI examinations, which had a magnetic field strength of 3T, at every 2-cycle interval. The imaging findings were independently evaluated and reported by 2 experienced radiologists.

### Treatment Regimen

#### NAT

All patients received NAT consisting of chemotherapy combined with targeted therapy. The targeted agents included trastuzumab with either pertuzumab or pyrotinib, administered for a minimum of 4 cycles. NAT regimens were categorized as (1) chemotherapy combined with dual monoclonal antibodies (taxane plus carboplatin plus trastuzumab plus pertuzumab [TCbHP] or taxane plus trastuzumab plus pertuzumab [THP]) or (2) chemotherapy combined with trastuzumab and pyrotinib (anthracycline plus cyclophosphamide followed by taxane plus trastuzumab with concurrent pyrotinib [AC-TH plus pyrotinib], taxane plus carboplatin plus trastuzumab plus pyrotinib [TCbH plus pyrotinib]; or taxane plus trastuzumab plus pyrotinib (TH plus pyrotinib]).

The standard treatment cycle was defined as 21 d. Selection of regimens was guided by individual tolerability to maintain both safety and therapeutic effectiveness.

#### Surgical Plan

Surgery occurred within 4 wk after NAT, following strict indications for breast-conserving surgery and reconstruction, with patient preference considered; mastectomy was performed if neither option was suitable. Sentinel lymph node biopsy was routine for patients without pre-NAT axillary metastasis, whereas confirmed metastasis by sentinel lymph node biopsy or pre-NAT involvement required axillary lymph node dissection. For patients in whom internal mammary or supraclavicular node metastasis was confirmed before NAT, dissection decisions were made on the basis of surgical expertise and imaging results.

### Postoperative Pathologic Evaluation

Surgical specimens were processed for standard paraffin embedding and histologic analysis. Immunohistochemical staining was routinely performed to assess estrogen receptor (ER), progesterone receptor (PR), HER2, and Ki-67 status. Tumors were considered HER2-positive if they had an immunohistochemical score of 3+ or an immunohistochemical score of 2+ with positive fluorescence in situ hybridization (FISH) confirmation. Pathologic response after NAT was assessed using both the Miller and Payne system and the residual cancer burden index. All pathologic assessments were independently performed by 2 experienced pathologists who were blinded to the imaging findings.

### Statistical Analysis

All analyses were conducted using Prism 10.1.2 (GraphPad Software). Normality of continuous variables was assessed with the Shapiro–Wilk test. Normally distributed data were compared using either the independent samples *t*-test or the Welch-corrected *t*-test, whereas nonnormally distributed data were evaluated with the Mann–Whitney *U* test. Categoric variables were examined using the χ^2^ test or Fisher exact test. Receiver-operating-characteristic curves were generated to estimate diagnostic performance, and the area under the curve (AUC) was calculated. Youden index was applied to identify the optimal cutoff value.

## RESULTS

### Patient Characteristics

A cohort of 45 patients diagnosed with HER2-positive breast cancer was initially recruited. During follow-up, 1 individual developed hepatic metastasis, and the corresponding data were excluded from subsequent analyses. The evaluable population comprised 44 patients (median age, 54 y; range, 29–73 y) (Table 1).

**TABLE 1.** Patient Characteristics (*n* = 44)

Characteristic	Value
Age (y)	
≤40	6 (14)
>40	38 (86)
Menopausal status	
Postmenopausal	30 (68)
Premenopausal	14 (32)
T stage	
T1	10 (23)
T2	23 (52)
T3	4 (9)
T4	7 (16)
N stage	
N0	8 (18)
N1	22 (50)
N2	4 (9)
N3	10 (23)
Clinical stage	
IIA	12 (27)
IIB	11 (25)
IIIA	5 (11)
IIIB	6 (14)
IIIC	10 (23)
ER positive	
≤10%	36 (82)
>10%	8 (18)
PR positive	
≤20%	41 (93)
>20%	3 (7)
HER2 IHC	
HER2 3+	42 (95)
HER2 2+, FISH positive	2 (5)
NAT regimen	
Macromolecular monoclonal antibody (trastuzumab plus pertuzumab)	21 (48)
Macromolecule–small-molecule combination (trastuzumab + pyrotinib)	23 (52)
Breast surgical procedure	
Breast-conserving surgery	5 (11)
Mastectomy	39 (89)
Lymph node surgical procedure	
SLNB	7 (16)
Non-SLNB	37 (84)
Postoperative pathology	
pCR	31 (70)
Non-pCR	13 (30)

IHC = immunohistochemistry; SLNB **=** sentinel lymph node biopsy.

Data expressed as number, followed by percentage in parentheses.

In this study, 21 patients (48%) received chemotherapy in combination with dual monoclonal antibodies, and 23 (52%) received chemotherapy combined with both large- and small-molecule targeted agents. Among them, 14 patients (32%) were treated with TCbHP, 5 (11%) with THP, 15 (34%) with AC-TH plus pyrotinib, 5 (11%) with TCbH plus pyrotinib, and 2 (5%) with TH plus pyrotinib. During treatment, 3 patients required regimen modification because of intolerance to adverse effects of AC-TH plus pyrotinib; 2 transitioned to the THP regimen and 1 to the TH plus pyrotinib regimen.

All 44 patients completed NAT followed by surgery, resulting in 31patients (70%) with pCR and 13 (30%) without pCR. No significant differences in pCR rates were observed across ages at diagnosis, menstrual status, clinical stage, ER expression, PR expression, Ki-67 index, NAT regimen, type of breast surgery, or lymph node procedure. In contrast, HER2 status demonstrated a statistically significant association with pCR. Specifically, 42 patients (95%) had an immunohistochemical score of HER2 3+, whereas 2 (5%) were HER2 2+ and FISH-positive; both HER2 2+/FISH-positive cases did not achieve pCR (Table 2).

**TABLE 2.** Characteristics of pCR and Non-pCR Groups (*n* = 44)

	Treatment response group			
Characteristic	pCR (*n* = 31)	Non-pCR (*n* = 13)	χ^2^	*P*
Age (y)			0.554	0.457
≤40	5 (83)	1 (17)		
>40	26 (68)	12 (32)		
Menopausal status			0.009	0.923
Postmenopausal	21 (70)	9 (30)		
Premenopausal	10 (71)	4 (29)		
Clinical stage			1.411	0.235
II	18 (78)	5 (22)		
III	13 (62)	8 (38)		
ER positive			1.965	0.161
≤10%	27 (75)	9 (25)		
>10%	4 (50)	4 (50)		
PR positive			0.022	0.882
≤20%	29 (71)	12 (29)		
>20%	2 (67)	1 (33)		
HER2 IHC			4.996	0.025
HER2 3+	31 (74)	11 (26)		
HER2 2+, FISH positive	0	2 (100)		
Ki-67 index			0.524	0.469
≤30%	13 (65)	7 (35)		
>30%	18 (75)	6 (25)		
NAT regimen			0.018	0.892
Macromolecular monoclonal antibody (trastuzumab plus pertuzumab)	15 (71)	6 (29)		
Macromolecule–small-molecule combination (trastuzumab plus pyrotinib)	16 (70)	7 (30)		
Breast surgical procedure			0.247	0.619
Breast-conserving surgery	4 (80)	1 (20)		
Mastectomy	27 (69)	12 (31)		
Lymph node surgical procedure			0.931	0.335
SLNB	6 (86)	1 (14)		
Non-SLNB	25 (68)	12 (32)		

IHC = immunohistochemistry; SLNB **=** sentinel lymph node biopsy.

Data expressed as number, followed by percentage in parentheses.

### Relationship Between ^68^Ga-HER2 PET/CT SUV_max_ and pCR

SUV_max_ of breast primary lesions at PET1 showed no significant difference between the pCR and non-pCR groups. In contrast, SUV_max_ at PET2 and PET3 were markedly reduced in the pCR group compared with the non-pCR group (PET 2: 0.45 ± 0.71 vs. 2.01 ± 2.36, *P* = 0.008; PET3: 0.12 ± 0.39 vs. 1.67 ± 2.94, *P* = 0.014). For lymph node metastases, SUV_max_ at PET1 remained comparable between groups, whereas SUV_max_ at PET2 and PET3 were lower in the pCR group (PET 2: 0.24 ± 0.45 vs. 2.49 ± 5.86, *P* = 0.113; PET3: 0.05 ± 0.22 vs. 1.00 ± 2.13, *P* = 0.107), although the differences did not reach statistical significance. (Table 3; Fig. 2).

**TABLE 3.** SUV_max_ of ^68^Ga-HER2 PET/CT Between pCR and Non-pCR Groups

Variable	pCR	Non-pCR	*P*
Breast primary lesions			
SUV_max_1	3.76 ± 1.97 (*n* = 30)	3.79 ± 2.71 (*n* = 13)	0.349
SUV_max_2	0.45 ± 0.71 (*n* = 28)	2.01 ± 2.36 (*n* = 12)	0.008
SUV_max_3	0.12 ± 0.39 (*n* = 21)	1.67 ± 2.94 (*n* = 7)	0.014
△SUV_max_1 (%)	−87 ± 20 (*n* = 27)	−60 ± 31 (*n* = 12)	0.007
△SUV_max_2 (%)	−95 ± 16 (*n* = 20)	−72 ± 33 (*n* = 7)	0.010
Lymph node metastases			
SUV_max_1	3.28 ± 2.11 (*n* = 25)	2.92 ± 2.94 (*n* = 10)	0.274
SUV_max_2	0.24 ± 0.45 (*n* = 24)	2.49 ± 5.86 (*n* = 9)	0.113
SUV_max_3	0.05 ± 0.22 (*n* = 17)	1.00 ± 2.13 (*n* = 6)	0.107
△SUV_max_1 (%)	−91 ± 19 (*n* = 22)	−59 ± 62 (*n* = 8)	0.130
△SUV_max_2 (%)	−98 ± 8 (*n* = 15)	−87 ± 23 (*n* = 5)	0.097

**FIGURE 2.**
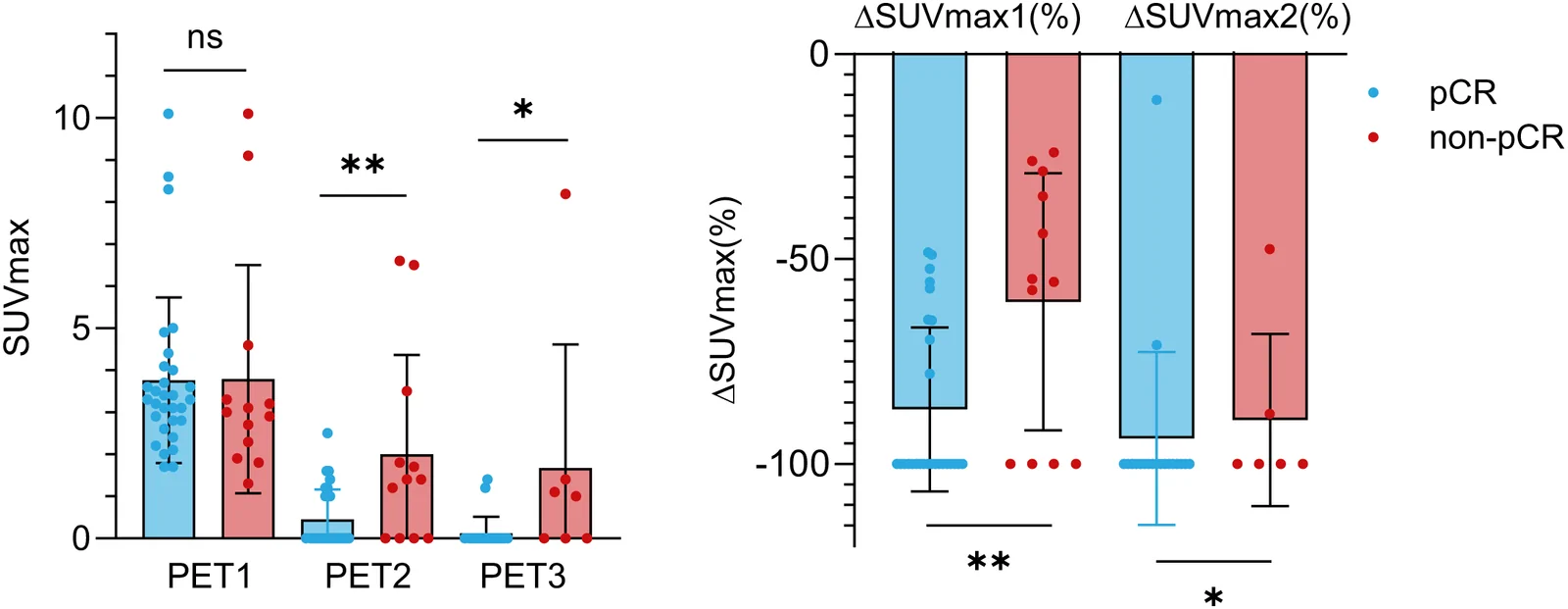
Comparison of SUV_max_ and ΔSUV_max_ in breast primary lesions between pCR and non-pCR groups.

Representative images in Figure 3 show the sequential ^68^Ga-HER2 PET/CT scans of a patient with HER2-positive breast cancer obtained before NAT, after the second treatment cycle, and before surgery.

**FIGURE 3.**
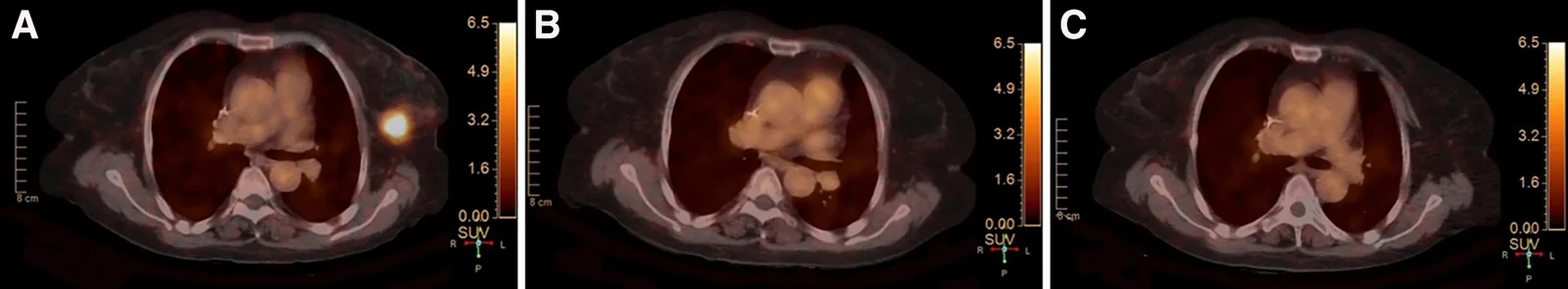
^68^Ga-HER2 PET/CT images before NAT, at end of second cycle, and before surgery. (A) Baseline scan demonstrated soft-tissue lesion measuring approximately 2.3 × 2.2 cm in upper outer quadrant of left breast, with marked radiotracer uptake and SUV_max_ of 10.1. (B) At end of second cycle, imaging revealed residual lesion of approximately 2.2 × 1.0 cm in same region, without abnormal radiotracer accumulation. (C) Preoperative imaging demonstrated further reduction in lesion size to approximately 2.0 × 0.6 cm, with no abnormal tracer uptake detected.

### Diagnostic Efficacy of ^68^Ga-HER2 PET/CT and Conventional MRI in Assessing NAT Response in HER2-Positive Breast Cancer

Receiver-operating-characteristic analysis was applied to evaluate the predictive value of SUV_max_, ΔSUV_max_1, and ΔSUV_max_2 in breast primary lesions at PET2 and PET3 for pCR. Sensitivity, specificity, PPV, and NPV were calculated according to the optimal cutoff thresholds, with detailed outcomes presented in Table 4 and Figure 4. The optimal cutoff values for pCR prediction included an SUV_max_ of no more than 1.15 (AUC = 0.738) at the end of the second NAT cycle and no more than 0.50 (AUC = 0.735) before surgery, as well as a ΔSUV_max_ of at least 61% (AUC = 0.742) at the end of the second cycle and at least 72% (AUC = 0.743) preoperatively.

**TABLE 4.** Concordance Between ^68^Ga-HER2 PET/CT SUV_max_ Cutoff Values and pCR Diagnosis

Variable	Cutoff	AUC	Sensitivity	Specificity	PPV	NPV	95% CI	Youden index
SUV_max_2	1.15	0.738	79	67	85	57	0.549–0.927	0.46
SUV_max_3	0.50	0.735	90	57	86	67	0.493–0.976	0.47
△SUV_max_1 (%)	−61	0.742	81	67	85	62	0.552–0.933	0.48
△SUV_max_2 (%)	−72	0.743	95	57	86	80	0.498–0.987	0.52

**FIGURE 4.**
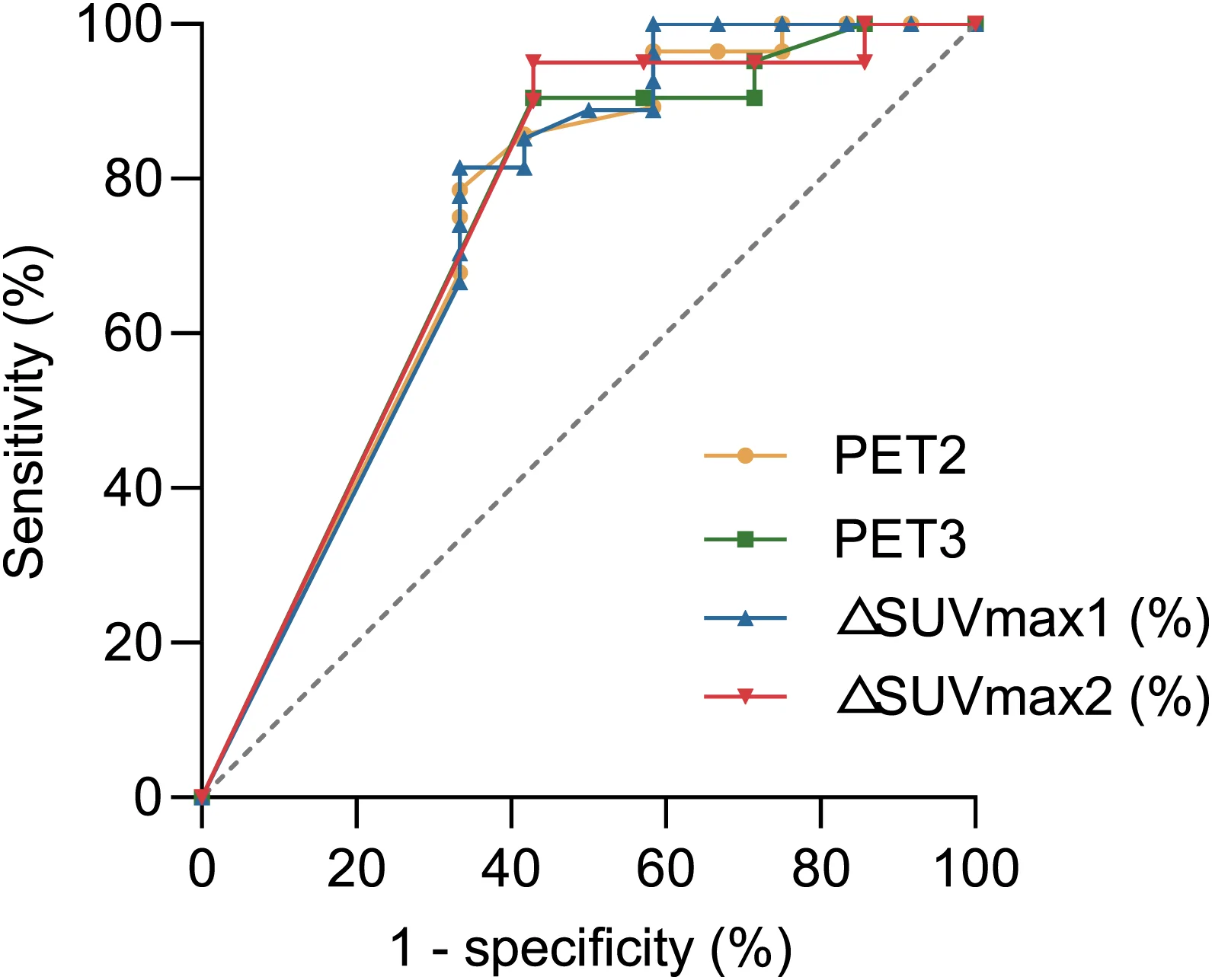
Receiver-operating-characteristic curves for predicting pCR using SUV_max_ and ΔSUV_max_ of breast primary lesions.

The diagnostic performance of MRI-defined complete response (per RECIST) for predicting pCR is provided in Table 5.

**TABLE 5.** Predictive Value of MRI-RECIST Complete Response for pCR

Variable	Sensitivity	Specificity	PPV	NPV	Youden index
At end of second therapy cycle	4	100	100	27	0.04
Before surgery	21	91	86	31	0.12

## DISCUSSION

With NAT’s growing use in the treatment of breast cancer, noninvasive, reproducible, and targeted strategies for predicting and monitoring therapeutic response are increasingly emphasized. Given PET/CT’s high diagnostic accuracy, its application has expanded to treatment evaluation. Our study investigated the clinical value of ^68^Ga-HER2 PET/CT for assessing NAT response in HER2-positive breast cancer.

As a HER2-targeted molecular imaging probe, ^68^Ga-HER2 binds to a different site on tumor cell HER2 receptors than do anti-HER2 drugs, with no competitive interaction. Consequently, a reduction in viable HER2-expressing tumor cell activity or density leads to decreased probe uptake. This provides the basis for using ^68^Ga-HER2 PET/CT to assess treatment efficacy, which is supported by the findings of the present study.

As a target-specific imaging biomarker, ^68^Ga-HER2 PET/CT directly probes the expression and availability of the HER2 receptor.​ This allows for the noninvasive monitoring of responses to HER2-targeted therapy, potentially earlier than when anatomic changes become apparent on conventional imaging. In this study, ^68^Ga-HER2 PET/CT predicted pCR in HER2-positive breast cancer both at an early stage (end of the second cycle of NAT) and at a later stage (preoperatively). PET2 SUV_max_ and ΔSUV_max_1 functioned as predictive markers for pCR. Moreover, ^68^Ga-HER2 PET/CT enabled early identification of HER2-positive breast cancer patients unlikely to benefit from NAT, thereby supporting timely modifications of therapeutic regimens or early surgical intervention to minimize unnecessary toxicity and financial burden. Such early prediction not only strengthens treatment confidence but also contributes to more individualized and precise management strategies, ultimately improving patient outcomes. Application of optimal cutoff thresholds for PET2 SUV_max_ and ΔSUV_max_1 on ^68^Ga-HER2 PET/CT enabled identification of patients responsive to NAT at the end of the second treatment cycle, with these patients demonstrating a postoperative pCR rate of 85%, representing a 15% higher likelihood of obtaining pCR compared with patients not assessed by this method. Whether modification of therapeutic regimens enhances pCR rates in nonresponders remains to be clarified. The presurgical ^68^Ga-HER2 PET/CT (PET3) provided significant predictive value for pCR, with SUV_max_ and ΔSUV_max_2 serving as potential indicators. Beyond prediction, the target-specific delineation of residual HER2-positive disease by PET3 could offer valuable complementary information in the surgical context.​ For instance, a complete response on PET3 may, in principle, increase surgical confidence when considering breast-conserving therapy, whereas precise localization of residual disease could aid in determining the extent of surgical resection. It is critical to acknowledge that the present study was designed as a diagnostic accuracy study and did not prospectively evaluate changes in surgical management on the basis of PET3 findings.​ Therefore, the potential of PET3 to guide the surgical approach remains a hypothesis derived from its imaging capabilities, which is grounded in the probe’s ability to accurately map residual tumor burden and warrants validation in future clinical trials.

PET metrics in primary tumors effectively predicted pCR but not in lymph nodes, potentially attributable to (1) biologic heterogeneity between sites (distinct molecular and microenvironmental characteristics), (2) divergent therapeutic responses (uneven drug delivery or immune infiltration), and (3) limited sample size, warranting further validation in larger cohorts.

MRI remains the standard of care, with well-established predictive value, having demonstrated 87% (147/169) PPV for pCR in hormone receptor (HR)–negative/HER2-positive breast cancer and 53% (73/138) PPV in HR-positive/HER2-positive cases in the TRAIN-3 trial (14). However, in the PHERGain trial, breast MRI complete response demonstrated a superior PPV in patients with HR-negative/HER2-positive (85.5%) breast cancer versus HR-positive/HER2-positive (61.5%) disease (15). In the present study, the PPV of presurgical MRI for pCR was comparable with that reported in previous studies. Our data indicated that preoperative ^68^Ga-HER2 PET/CT achieved a PPV of 86%, regardless of HR status, when ΔSUV_max_2 was used as the evaluation criterion. The advantage of ^68^Ga-HER2 PET/CT is its higher overall diagnostic performance, as evidenced by a higher Youden index for predicting pCR in the HER2-positive subtype, whereas limited clinical accessibility remains its principal disadvantage.

Traditional ^18^F-FDG PET/CT has been widely investigated for assessing the efficacy of NAT in breast cancer. Humbert et al. reported that after the first treatment cycle, the predictive accuracy of pCR on the basis of changes in SUV_max_ varied across subtypes, with AUCs of 0.82 for triple-negative, 0.65 for HER2-positive, and 0.90 for luminal-type breast cancers (16). Subsequent evidence indicated that SUV_max_ measured at the end of the second treatment cycle provided the most reliable parameter for evaluating therapeutic response (17,18). In the PHERGain trial, ^18^F-FDG PET/CT demonstrated a higher PPV in HR-positive/HER2-positive (82.0%) versus HR-negative/HER2-positive (75.0%) cases (15). Chen et al. examined the performance of ^68^Ga-FAPI PET/CT in a cohort of 22 patients, of whom 7 (31.8%) achieved pCR and 15 (68.2%) did not (19). A reduction of 63.8% in SUV_max_ at the end of the second cycle yielded the strongest predictive value for pCR (AUC = 0.879). The distinct tracers and imaging mechanisms of ^18^F-FDG, ^68^Ga-FAPI, and ^68^Ga-HER2 PET/CT result in varying diagnostic performance for therapy monitoring. However, limited sample sizes in current studies may introduce bias, and overall research remains insufficient, warranting further investigation.

Table 4 illustrates the suboptimal specificity and NPV for the ^68^Ga-HER2 PET/CT SUV_max_ cutoff, indicating potential undertreatment risks. We acknowledge this limitation and propose a strategy to enhance predictive accuracy. Our ongoing research integrates ^68^Ga-HER2 PET with minimal residual disease detection to refine therapeutic decision-making. Specifically, some patients may achieve imaging-based negativity during NAT yet remain minimal residual disease–positive, indicating residual tumor cells undetectable by conventional methods. For such cases, treatment escalation—rather than de-escalation—may be warranted to mitigate undertreatment risks associated with imaging-alone false negatives. Recent studies have demonstrated the utility of composite biomarkers, combining PET imaging with tissue-based assays to improve PPV and NPV. For instance, Llombart-Cussac et al. demonstrated that the HER2DX assay significantly predicts pCR in HER2-positive breast cancer, independent of chemotherapy regimen (20), whereas Hennessy et al. reported that a composite biomarker (C1D15 SULmax PET metrics plus HER2 protein abundance) achieved superior sensitivity (100%) and NPV (100%) (21).

Previous research has identified ER/PR status as a predictor of pCR in HER2-positive breast cancer, although our study did not confirm this correlation. According to National Comprehensive Cancer Network guidelines, our cohort received similar NAT regimens for HER2-positive and luminal B/HER2-positive subtypes. The literature suggests better pCR rates in pure HER2-positive tumors, possibly attributable to ER/HER2 cross-talk, where ER activation may diminish HER2-targeted therapy efficacy via survival pathways like PI3K/AKT/mTOR (2). However, our data showed no significant difference in ER/PR status between pCR (*n* = 31) and non-pCR (*n* = 13) groups, possibly because of the limited sample size.

Extensive investigation in the neoadjuvant setting of HER2-positive breast cancer has focused on strategies to enhance pCR outcomes. The NeoSphere trial reported a 45.8% pCR rate with the THP regimen, establishing dual-target therapy as a reference standard for HER2-positive NAT (22). The PEONY study further demonstrated a pCR rate of 39.3% for the same regimen in Asian populations, closely mirroring results from the NeoSphere trial (23). In the TRAIN-2 trial, a regimen of pertuzumab plus trastuzumab plus paclitaxel plus carboplatin achieved a markedly higher pCR rate of 68% (24). In the PHEDRA trial, the pCR rate with TH plus pyrotinib reached 41.0% (25). In the present cohort, 10 patients were diagnosed with clinical stage IIIC disease (23%), and the postoperative pathologic pCR rate was 70%, a result consistent with previous evidence but slightly higher than previously reported rates, possibly reflecting differences in NAT regimen selection. Among our 44 patients, 21 (48%) received chemotherapy combined with dual monoclonal antibodies, whereas 23 (52%) were treated with chemotherapy combined with large- and small-molecule targeted agents. The higher pCR rate observed in our cohort may be attributable to the use of combination anti-HER2 therapy. The complementary mechanisms of monoclonal antibodies (extracellular blockade) and small-molecule tyrosine kinase inhibitors (intracellular kinase inhibition) are thought to provide a more comprehensive suppression of HER2 signaling, potentially overcoming de novo or acquired resistance and leading to deeper pathologic responses (26,27).

Several limitations should be noted. First, the small sample size and single-center design limit generalizability. Further, the heterogeneous NAT regimens may have confounded SUV_max_ and pCR outcomes. Also, the absence of follow-up data limits long-term survival prediction; the lack of ^18^F-FDG PET/CT comparison reduces comprehensiveness, and the ability to differentiate benign from malignant lesions remains unverified. Finally, the safety of the ^68^Ga-HER2 PET/CT needs validation in larger trials, although no adverse events were observed.

This study provide preliminary evidence supporting the application of ^68^Ga-HER2 PET/CT in assessing therapeutic efficacy during NAT of HER2-positive breast cancer. Validation in larger cohorts remains essential to confirm reliability and enhance clinical generalizability. Future investigations may consider performing ^68^Ga-HER2 PET/CT at the end of the second treatment cycle to identify patients unlikely to achieve pCR, allowing timely modification of therapeutic strategies to potentially improve pCR rates and overall outcomes.

## CONCLUSION

^68^Ga-HER2 PET/CT enables assessment of early alterations in tracer uptake during NAT in HER2-positive breast cancer and demonstrates predictive value for pCR. The ability to predict pathologic response at both early treatment cycle and preoperative stages highlights its potential as a clinical tool for guiding individualized treatment strategies.

## DISCLOSURE

This work was supported by the S&T Program of Hebei (242W7701Z). No other potential conflict of interest relevant to this article was reported.

## Notes

KEY POINTS **QUESTION:** In patients with biopsy-confirmed HER2-positive breast cancer, can early assessment with ^68^Ga-HER2 PET/CT during NAT predict pCR after treatment? **PERTINENT FINDINGS:** This prospective cohort study analyzed 44 patients and demonstrated that a reduction in primary tumor SUV_max_ of at least 61% after the second treatment cycle was associated with a pCR rate of 85%. In contrast, patients with a reduction of less than 61% achieved a pCR rate of only 38%, a difference that was statistically significant. **IMPLICATIONS FOR PATIENT CARE:** Early ^68^Ga-HER2 PET/CT enables the rapid identification of nonresponders, allowing for timely adaptation of the NAT strategy, such as switching regimens or expediting surgery. This approach mitigates unnecessary treatment-related toxicity and financial burden, thereby facilitating personalized, precision oncology.
